# Temporal and electroencephalography dynamics of surreal marketing

**DOI:** 10.3389/fnins.2022.949008

**Published:** 2022-10-27

**Authors:** Regina W. Y. Wang, I-Ning Liu

**Affiliations:** ^1^Department of Design, National Taiwan University of Science and Technology, Taipei City, Taiwan; ^2^Design Perceptual Awareness Laboratory, Taiwan Building Technology Center, National Taiwan University of Science and Technology, Taipei City, Taiwan

**Keywords:** visual language, metaphor, curiosity, purchase intention, event-related spectral perturbation (ERSP)

## Abstract

Event-related spectral perturbation analysis was employed in this study to explore whether surreal image designs containing metaphors could influence product marketing effects, including consumers’ product curiosity, product comprehension, product preference, and purchase intention. A total of 30 healthy participants aged 21–30 years were recruited. Neurophysiological findings revealed that lower gamma, beta, and theta spectral powers were evoked in the right insula (Brodmann Area 13) by surreal marketing images. This was associated, behaviorally, with the manifestation of higher product curiosity and purchase intention. Based on previous research, the brain functions of this area include novelty, puzzle-solving, and cravings for reward caused by cognitive overload.

## Introduction

René Magritte, a surrealist master who became renowned as a painter-philosopher, once said, “Everything we see hides another thing, we always want to see what is hidden by what we see (Lipinski, 2019).” Surreal marketing images are a type of visual metaphor. The creation of images that surpass the average imagination for the purposes of promoting brand products is a form of creative visual marketing strategy (Othman, 2021). This approach involves the use of dreamlike and imaginative images to forge deep impressions of branded products (Mostafa, 2005). Some marketers promote their products based purely on function and design, whereas others are not limited by these aspects, opting instead for novel images with irrational and plural metaphorical sources which create an overarching narrative or theme, more memorable to an audience (Peterson et al., 2017). An example of this is the marketing campaign image employed in the product launch of the Chanel Chance Eau Vive fragrance (Chanel, 2017). To appeal to a younger, popular market, surreal marketing images were used in which perfume bottles were transformed into bowling balls that could be bowled by female models in a bowling alley under a fantastical sky full of stars. Another example is a series of surreal marketing campaign images released by Volkswagen for a new line of cars (Feeldesain, 2015). Instead of viewing images of the new cars, the audience is triggered into forming mental associations with a surrealist content which includes the bizarre merging of a tiger’s head and a bee’s body, thereby conveying the product concept that the new cars are as powerful as tigers but as light as bees. Visual metaphors tend to arouse the curiosity of viewers (Najmuldeen, 2015), so that consumers take the initiative to learn about a new product (Ashraf et al., 2019). The question of whether surreal visual language can be used in a commercial product launch is one that is worth exploring, and requires further investigation of consumer demands and the stages of internal consumer responses, ranging from curiosity, comprehension and preference to purchase intention (Kotler and Armstrong, 1980; Grunert and van Trijp, 2014; Wijaya, 2015; Ashraf et al., 2019).

Currently, the tools used in neuroscience research on consumers include Electroencephalography (EEG), Functional Magnetic Resonance Imaging (fMRI), Functional Near-Infrared Spectroscopy (fNIRS), Electrocardiogram (ECG), Eye Tracking (ET), Galvanic Skin Response (GSR) and Facial Expression Recognition Software (fERS). EEG, compared with other tools, is non-invasive, with low equipment and testing costs. It has been the most commonly used neuroscience tool in neuromarketing research in recent years (Alvino et al., 2020). EEG can detect brain potential changes at the scale of milliseconds, thus developing a complete representation of the participant’s cognitive and physiological responses (Morin, 2011; Vecchiato et al., 2011). Since the first human EEG recording by Hans Berger in the 1920s, this technique has been widely used (Herrmann et al., 2016; Bazzani et al., 2020). It is one of several neuroscience tools that may assist with the examination of the brain responses of consumers to specific marketing elements. In contrast to more traditional methods of marketing research (e.g., questionnaire surveys and behavioral performance), EEGs monitor the real-time and continuous acquisition of information by participants (Bazzani et al., 2020). The high temporal resolution of an EEG means it can capture brain activity almost at the speed of cognition (Kalaganis et al., 2021). Several multinational companies such as Coca-Cola, Google, and Disney have employed neuromarketing research to measure the effectiveness of their products (Morin, 2011; Ienca and Andorno, 2017). Neuromarketing is an interdisciplinary research field (Robaina-Calderín and Martín-Santana, 2021). According to relevant neuromarketing studies, the activation of the insula and medial prefrontal cortex are both associated with product purchasing decisions (Tusche et al., 2010). Participants who viewed ambiguous images that elicited their curiosity showed activations in their anterior insula and anterior cingulate cortex (ACC) (Jepma et al., 2012). Many other neuroscientific studies in relevant art fields have revealed that the cognitive conflict and memories inspired by surreal images produce relatively stronger theta activity in the center of the frontal lobe (Ruzzoli et al., 2020). Humorous drawings elicit theta activity in the parietal lobe and posterior cingulate cortex (Wang et al., 2017). Compared with non-award-winning advertisements, it has been found that award-winning advertisements that combine artistic aesthetics with marketing elicit greater theta and alpha activity in the right frontotemporal regions (Wang et al., 2018b). The abovementioned relevant cross-disciplinary work is not limited to the field of marketing, but includes also fields in the visual arts such as painting, graphic design, advertising creativity, and neural networks in artificial intelligence. This study employed EEG tools and ERSP analyses (Delorme and Makeig, 2004) to examine whether surreal product images could impact marketing effectiveness, that is, how different internal consumer responses (curiosity, comprehension, preferences, and purchase intention) are reflected in EEG spectral perturbations.

Surrealism was a major avant-garde movement of the 20*^th^* century (Mostafa, 2005). The surrealist artists of that time were heavily influenced by Sigmund Freud’s literary masterpiece, *The Interpretation of Dreams*, which meant that many of their creative inspirations came from dreams and imagination (Jiménez et al., 2013). One feature of surrealist paintings is the combination of unrelated images, in which impossible layouts are used to establish irrational connections between images to convey specific metaphors (Forceville, 1988; Wade, 2015), thus giving rise to strangeness, dissonance, and ambiguity (Furnham and Avison, 1997). The activation of the precuneus is associated with viewing reality-distorting stimuli, such as when participants are asked to watch movie clips from the fantasy film Alice in Wonderland, including a scene where Alice talks to the floating, disembodied head of the Cheshire cat, which then starts to spin and grow a body (Rikandi et al., 2017). The word “metaphor” is derived from the Greek word “metaphorá” (transference) and refers to the transfer of meaning from one object to another, thereby achieving the transmission of meaning between different domains (Garner, 2005). Surreal images do not simply convey concepts through one metaphorical source but will do so by combining two or more metaphorical sources (Najmuldeen, 2015). For example, the classic painting by René Magritte, *The Son of Man* (Magritte, 1964), contains within it several metaphorical sources. It depicts a man wearing a black bowler hat and overcoat, with his facial features largely obscured by a hovering green apple (metaphorical source 1), but with half an eye peeking over the edge of the apple (metaphorical source 2). His left arm appears to be unreasonably bent backward at the elbow (metaphorical source 3), and he is standing before the sea and a cloudy sky (metaphorical source 4). The apple in the painting can be interpreted as the temptation of Adam and the fall of man in the Garden of Eden. The obscured facial features with only half an eye exposed represents how mortals can only peep at the truth. The left elbow bent backward is a metaphor for the irrational manner by which the world operates, and the cloudy sky and sea allude to the world of uncertainty in which mortals exist (Knauss, 2015; Waligorska-Olejniczak, 2018).

We propose the following hypothesis: Surreal marketing images with a greater number of metaphorical sources will evoke a higher spectral power in association with internal consumer responses (product curiosity, product comprehension, product preference, and purchase intention). This hypothesis equates the process of interpreting metaphors to solving a puzzle, which is a pleasurable experience for consumers (Phillips and McQuarrie, 2004). When consumers succeed at solving a puzzle, the resulting feelings of pleasure are transferred to the product to create positive evaluations (Peracchio and Meyers-Levy, 1994). However, comprehension is a necessary prerequisite for experiencing pleasure, and a puzzle that is too difficult for the viewer to solve will produce feelings of confusion and frustration instead (Stafford et al., 1996; Van Mulken et al., 2014), causing a negative evaluation of the product.

**FIGURE 1:**
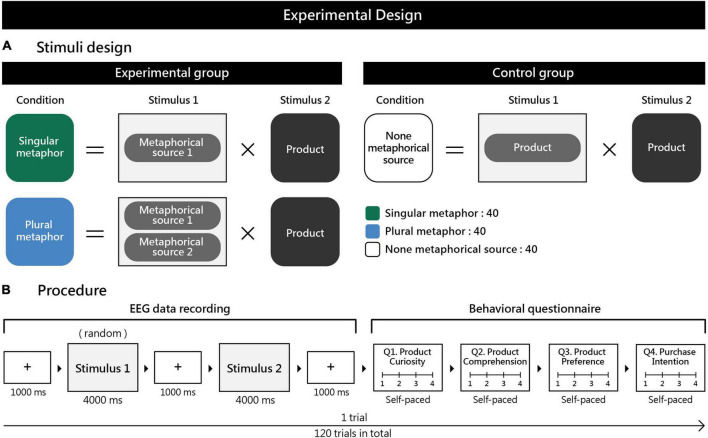
**(A)** Each experimental condition consisted of stimuli 1 and 2. The three conditions are described as follows: The singular metaphorical source condition involved presenting a surreal marketing image with one metaphorical source (stimulus 1) + an actual product image (stimulus 2), for a total of 40 trials. The plural metaphorical sources condition involved presenting a surreal marketing image with two metaphorical sources (stimulus 1) + an actual product image (stimulus 2) for a total of 40 trials. The no metaphorical source condition involved presenting actual product images for both stimuli 1 and 2 for a total of 40 trials. **(B)** One trial in the experimental procedure consisted of EEG data recording and the administration of a behavioral questionnaire. EEG data recording: A fixation cross was first presented for 1000 ms, followed by the randomized presentation of marketing images (stimulus 1) for 4000 ms. The fixation cross was presented again for 1000 ms, and then the product image (stimulus 2) was presented for 4000 ms. At the end of the EEG data recording, the behavioral questionnaire was administered. Participants were required to answer questions on product curiosity, comprehension, preference, and purchase intention using a four-point Likert scale (1 = completely disagree, 2 = disagree, 3 = agree, 4 = completely agree). The questionnaire response time was self-paced. The entire experiment consisted of 120 trials and lasted for 30–40 min.

In a pilot survey of one of the world’s largest creative advertising communities, Ads of the World (Clio Network, 2017), we found that most practical print advertisements contained one or two metaphorical sources, whereas advertisements with more than two metaphorical sources were uncommon. Studies related to metaphorical advertising mostly examined images with one or two metaphorical sources (Phillips and McQuarrie, 2004; Van Mulken et al., 2010; Forceville et al., 2016). In this study, images were divided into singular and plural metaphorical sources, with the addition of a control group (no metaphorical source) (Figure 1A). Singular metaphorical sources refer to surreal marketing images with only one metaphorical source alluding to the product. Plural metaphorical sources refer to surreal marketing images with two metaphorical sources alluding to the product. The control group refers to non-surreal images without any metaphorical source that conveys the design and function of the product through actual product images. High-density EEG (Liu et al., 2017; Seeber et al., 2019) was performed to explore the effects of surreal marketing images with different metaphorical sources on brain responses (Figure 1B).

## Materials and methods

### Participants

A total of 30 healthy participants (14 males, 16 females) with bachelor’s or master’s degrees, aged 21–30 years (mean age = 24.6 years), were recruited. All participants had a corrected visual acuity of above 0.8 and did not have color blindness, visual impairments, a medical history of neurological or mental disorders, and drug or alcohol addiction. Participants were asked to stop using stimulants that might affect their EEG responses (e.g., alcohol, caffeine) 48 h prior to the experiment. This study was conducted in accordance with the Declaration of Helsinki (World Medical Association, 2001, 2013). Participants gave informed consent in writing before the experiment, and the study was approved by the Institutional Review Board of Cathay General Hospital.

### Stimuli

Surreal marketing images are frequently used in the design of print advertisements. In this study, we collected print advertisements released between 2005 and 2017 from one of the world’s largest creative advertising communities, Ads of the World (Clio Network, 2017). Photoshop software was used to remove brand and textual information from the print advertisements to produce simple marketing images. Prior to the experiment, a focus group consisting of five experts with design-related backgrounds were asked to identify the number of metaphorical sources in the marketing images and to classify these as conditions in respective groups. The design of these experimental conditions had to first confirm a proper association between stimulus 1 and stimulus 2 (Figure 1A). The metaphorical source image (stimulus 1) had to be applied and associated with the appropriate product (stimulus 2) in existing marketing campaigns (Clio Network, 2017). For example, the metaphorical image of the bizarre merging of a tiger’s head with a bee’s body (stimulus 1) was paired with Volkswagen’s new model (stimulus 2) (Ogilvy, 2015). The design conditions were described as follows: (i) a singular metaphorical condition was one metaphorical source marketing image paired with the appropriate product image; (ii) a plural metaphorical condition was two metaphorical source marketing images paired with the appropriate product image; and the control group of stimulus 1 or 2 was a non-metaphorical source marketing image, in which the product image spoke for itself.

### Procedure

This experiment was conducted in the Design Perceptual Awareness Lab (D:PAL) of the National Taiwan University of Science and Technology. Interference from external noise, temperature, and light were strictly controlled during the experimental process. Participants underwent an EEG recording and responded to a behavioral questionnaire in the lab, while the experimenter monitored the participant through a camera outside the lab and communicated with them using an intercom. A Neuroscan EEG recording and analysis system (Scan 4.3.3 & STIM2) was used in this experiment, consisting of a 64-channel EEG cap (64-channel Quik-Cap) and amplifier (SynAmps 2), which recorded EEG signals *via* 64 channels according to the International 10-10 system of electrode placement. The reference electrode was placed at the central parietal lobe (between Cz and CPz), and the ground electrode was placed on the forehead (AFz) (Neuroscan, 2022).

Participants viewed the stimuli on a 50*30 cm monitor, placed on a desk at a height of 74 cm and at a distance of approximately 60–70 cm. The center of the screen was within a visual angle of 10–20°. Before the start of the experiment, participants were first briefed on the purpose and procedure of the study. The experiment only proceeded after the participants had fully understood the content of the experiment and had provided their written consent. The procedure was as follows (Figure 1B): A fixation cross was first presented for 1000 ms on the screen, followed by the randomized presentation of surreal marketing images (stimulus 1) for 4000 ms. The fixation cross was then presented for 1000 ms, followed by the product image (stimulus 2) paired with stimulus 1 for 4000 ms. The fixation cross then appeared again for 1000 ms. Finally, the behavioral questionnaire was administered, including the following questions: (1) Does this image make you curious about the product? (2) Does this image give you a clear understanding of the product? (3) Does this image make you like the product? (4) Does this image make you want to buy the product? Responses were collected using a four-point Likert scale (1 = completely disagree, 2 = disagree, 3 = agree, 4 = completely agree). Participants responded by pressing the numbers on the keyboard, and the timing was self-paced. The above describes the procedure for one trial. The entire experiment consisted of 120 trials and lasted for 30–40 min.

### Behavioral data analyses

Descriptive statistics were employed to measure the independent variable (singular metaphorical source, plural metaphorical sources, no metaphorical source). One-way repeated-measures of ANOVA were performed to determine whether the independent variable led to significant differences in the mean scores of the dependent variables (product curiosity, comprehension, preference, and purchase intention). For significant results, LSD *post-hoc* comparisons were performed to verify the differences in the independent variables, and the eta-squared effect size was calculated (Cohen, 1988). To obtain the ERSP results for high/low levels of internal consumer responses, the responses to the behavioral questionnaire were used to divide the 120 stimuli into high/low product curiosity, comprehension, preference, and purchase intention. The cutoff point was defined as the median (2.5 points) of participants’ scores ranging from one to four points, whereby stimuli scoring greater and lower than 2.5 points were categorized as high and low, respectively (Table 1). Independent-sample one-way ANOVA was performed to determine whether there were significant differences in the mean scores of high/low-level stimuli.

**TABLE 1:** Mean scores and number of stimuli for high/low levels of internal consumer responses.

Stages of consumer response	Total number	Level	Mean	Number
Product curiosity	120 items	High product curiosity	3.12	88 items
		Low product curiosity	2.21	32 items
Product comprehension	120 items	High product comprehension	3.28	70 items
		Low product comprehension	1.89	50 items
Product preference	120 items	High product preference	2.79	68 items
		Low product preference	2.24	52 items
Purchase intention	120 items	High purchase intention	2.80	62 items
		Low purchase intention	2.21	58 items

### Independent components analysis and clustering

Independent components analysis (ICA) is a method involving the conversion of mixed signals into independent components. Since EEG signals are derived from a mixture of neural signal sources from various regions in the cerebral cortex, ICA can separate the EEG signals into several independent components and filter out noise (eye movements, muscle artifacts) from the signals (Rejer and Górski, 2015). ICA was performed using the MATLAB open-source EEG Lab toolbox (Delorme and Makeig, 2004) according to the following steps: (a) The segments for analysis (−1000 ms ∼ 4000 ms) were extracted from the continuously recorded EEG data. (b) The EEG signals were down-sampled to 250 Hz to reduce data storage and analysis time. (c) The finite impulse response filter was applied, with a high-pass filter value of 1 Hz and a low-pass filter value of 100 Hz. (d) ICA was implemented to separate the 64-channel signals into 64 ICs. (e) Using K-means clustering (MacQueen, 1967), 13 parcellated brain regions were demarcated, consisting of the left frontal, frontal midline, right frontal, left temporal, central midline, right temporal, left parietal, parietal, right parietal, left occipital, occipital midline, right occipital, and limbic system (f) Based on the equivalent dipole location and scalp map features of the 13 brain regions, the 1920 ICs (30 participants * 64 ICs) were grouped into 13 brain regions. ICs that did not match the required features were discarded, which meant that each brain region had a different number of ICs. (g) Finally, the Talairach x, y, z coordinates of the region centroids of each brain area were mapped to the corresponding BA to understand the actual brain functions of a given area (Garey, 1999; Thottakara et al., 2006).

### Event-related spectral perturbation analysis

Event-related spectral perturbations are the spectral perturbations in the brain evoked by stimuli. ERSP analysis was performed using the ERSP techniques available in the EEG Lab toolbox of MATLAB (Delorme and Makeig, 2004). The steps for ERSP analysis were: (a) Wavelet transformation (Mallat, 1989; Burrus et al., 1997) was performed to transform the EEG signals from each trial into time and frequency signals. (b) The signals were normalized according to the spectral power of the baseline. (c) The signals were averaged across all trials to obtain the ERSP images, in which the intensity of spectral perturbations was proportional to color brightness, and a darker color (e.g., a darker red or blue) implied a stronger spectral perturbation. Red, blue, and green denoted an increase, decrease, and no significant difference in spectral power compared to baseline, respectively (Pfurtscheller and Da Silva, 1999; Zhang and Gu, 2018). After obtaining the ERSP data, further research was conducted on the different frequency bands: gamma (γ, 31–100 Hz), beta (β, 14–30 Hz), alpha (α, 8–13 Hz), theta (θ, 4–7 Hz) and delta (δ, 1–3 Hz) (Herrmann et al., 2016; Wang et al., 2018a,^2020^). Paired sample *t*-tests were performed comparing the following experimental conditions: “high vs. low product curiosity,” “high vs. low product comprehension,” “high vs. low product preference,” “high vs. low purchase intention,” “singular vs. plural metaphorical sources,” “no vs. singular metaphorical source,” “no vs. plural metaphorical sources,” to determine whether there were significant differences in the spectral powers between the different conditions (*p* < 0.05).

## Results

### Behavioral results

**FIGURE 2:**
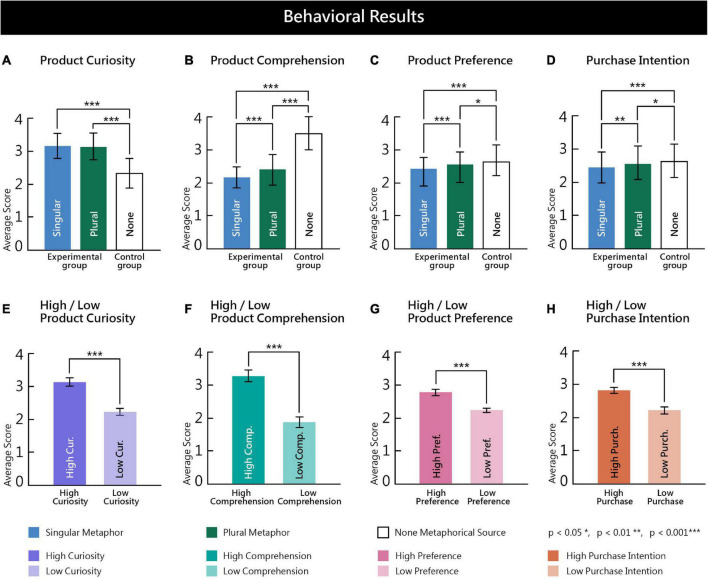
**(A–D)** The different number of metaphorical sources (independent variable) led to significant differences in product curiosity, comprehension, preference, and purchase intention (dependent variables) (*p* < 0.05). The product curiosity of the experimental groups was higher than that of the control group, whereas the product preference, comprehension, and purchase intention of the control group were higher than those of the experimental groups, while those of plural metaphorical sources were higher than those of singular metaphorical source. **(E–H)** Significant differences were observed between high- and low-level internal consumer responses, with high-level product curiosity, comprehension, preference, and purchase intention showing higher scores than low-level consumer responses (*p* < 0.001).

We hypothesized that surreal marketing images with more metaphorical sources would evoke higher levels of product curiosity, comprehension, preference, and purchase intention. A one-way repeated-measure analysis of variance (ANOVA) was performed to determine the effects of the independent variable on the dependent variables, and the eta-squared effect sizes were calculated (0.01 ≤ η^2^p small effect size < 0.06; 0.06 ≤ η^2^p moderate effect size < 0.14; 0.14 ≤ η^2^p large effect size) (Cohen, 1988). A total of 3,600 sample values (30 participants * 3 experimental conditions * 40 sample items) were analyzed. The statistical data of the behavioral results are shown below (Figures 2A–D). Surreal marketing images with different numbers of metaphorical sources showed significant differences in product curiosity (*F* = 454.637, df = 1.847, *p* = 0.000 < 0.001, η^2^p = 0.275). Least significant difference (LSD) *post-hoc* comparisons revealed that images with singular (*N* = 1200, *M* = 3.17, SD = 0.770) and plural metaphorical sources (*N* = 1200, *M* = 3.14, SD = 0.805) were both higher than the control group (*N* = 1200, *M* = 2.32, SD = 0.935; Figure 2A). Surreal marketing images with different metaphorical sources showed significant differences in product comprehension (*F* = 782.913, df = 1.954, *p* = 0.000 < 0.001, η^2^p = 0.395). LSD *post-hoc* comparisons revealed that images with plural metaphorical sources (*N* = 1200, *M* = 2.41, SD = 1.046) were higher than images with a singular metaphorical source (*N* = 1200, *M* = 2.18, SD = 0.962), while the control group (*N* = 1200, *M* = 3.51, SD = 0.653) was higher than both images with a singular metaphorical source (*N* = 1200, *M* = 2.18, SD = 0.962) and images with plural metaphorical sources (*N* = 1200, *M* = 2.41, SD = 1.046; Figure 2B). Surreal marketing images with different metaphorical sources showed significant differences in product preference (*F* = 24.244, df = 2.000, *p* = 0.000 < 0.001, η^2^p = 0.020). LSD *post-hoc* comparisons revealed that images with plural metaphorical sources (*N* = 1200, *M* = 2.57, SD = 0.962) were higher than images with a singular metaphorical source (*N* = 1200, *M* = 2.43, SD = 0.922), while the control group (*N* = 1200, *M* = 2.66, SD = 0.862) was higher than both images with a singular metaphorical source (*N* = 1200, *M* = 2.43, SD = 0.922) and images with plural metaphorical sources (*N* = 1200, *M* = 2.57, SD = 0.962; Figure 2C). Surreal marketing images with different metaphorical sources showed significant differences in purchase intention (*F* = 16.065, df = 2.000, *p* = 0.000 < 0.001, η^2^p = 0.013). LSD *post-hoc* comparisons revealed that images with plural metaphorical sources (*N* = 1200, *M* = 2.52, SD = 0.060) were higher than images with a singular metaphorical source (*N* = 1200, *M* = 2.41, SD = 1.001), while the control group (*N* = 1200, *M* = 2.61, SD = 0.960) was higher than both images with a singular metaphorical source (*N* = 1200, *M* = 2.41, SD = 1.001) and images with plural metaphorical sources (*N* = 1200, *M* = 2.52, SD = 0.060; Figure 2D). To summarize the behavioral results above, images with plural metaphorical sources were associated with higher product comprehension, product preference, and purchase intention compared to images with a singular metaphorical source (*p* < 0.001), but no significant difference was observed between the two groups in terms of product curiosity (*p* > 0.05). Images with no metaphorical sources led to higher product comprehension, preference, and purchase intention than images with singular or plural metaphorical sources (*p* < 0.05). However, both the singular and plural metaphorical source groups elicited a higher level of product curiosity compared to the control group with no metaphorical sources (*p* > 0.001). Validation of our hypothesis in terms of our behavioral findings will be discussed later in conjunction with the event-related spectral perturbation (ERSP) results obtained.

To examine the ERSP spectral power of markedly high levels of internal consumer responses, we analyzed whether there were significant differences between the high and low levels of each internal consumer response. Independent-sample one-way ANOVA was performed on the behavioral questionnaire which was included as part of the EEG study design. The cutoff point was defined as the median (2.5 points) of the participants’ scores, ranging from one to four points, such that stimuli scoring greater and lower than 2.5 points were categorized as high and low-level, respectively. There was a significant difference in the behavioral scores of stimuli with high/low product curiosity (*F* = 459.73, df = 1, *p* = 0.000 < 0.001). Stimuli with high product curiosity (*N* = 88, *M* = 3.12, SD = 0.21) inspired higher scores than those with low product curiosity (*N* = 32, *M* = 2.21, SD = 0.18; Figure 2E). A significant difference was observed in the behavioral scores of stimuli with high/low product comprehension (*F* = 499.18, df = 1, *p* = 0.000 < 0.001). Stimuli with high product comprehension (*N* = 70, *M* = 3.28, SD = 0.35) elicited higher scores than those with low product comprehension (*N* = 50, *M* = 1.89, SD = 0.32; Figure 2F). There was a significant difference in the behavioral scores of stimuli with high/low product preference (*F* = 294.91, df = 1, *p* = 0.000 < 0.001). Stimuli with high product preference (*N* = 68, *M* = 2.79, SD = 0.18) produced higher scores than those with low product preference (*N* = 52, *M* = 2.24, SD = 0.16; Figure 2G). Finally, there was a significant difference in the behavioral scores of stimuli with high/low purchase intention (*F* = 323.79, df = 1, *p* = 0.000 < 0.001). Stimuli with high purchase intention (*N* = 62, *M* = 2.80, SD = 0.18) were associated with higher scores than those with low purchase intention (*N* = 58, *M* = 2.21, SD = 0.19; Figure 2H). To summarize, all high-level internal consumer responses (product curiosity, comprehension, preference, and purchase intention) inspired higher scores than low-level internal consumer responses (*p* < 0.001). Further analysis should, however, be carried out with regard to the ERSP spectral power of high-level internal consumer responses.

### Event-related spectral perturbation results

**FIGURE 3:**
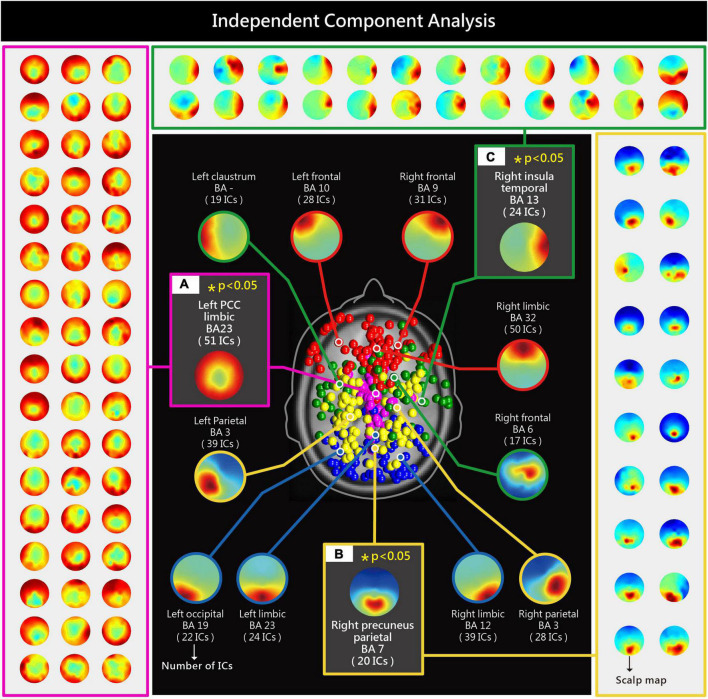
Analysis of the spectral power of high/low levels of internal consumer responses (product comprehension, curiosity, preference, purchase intention) and surreal marketing images with different numbers of metaphorical sources revealed significant differences (*p* < 0.05) in the following brain areas: left PCC **(A)**, right precuneus **(B)** and right insula **(C)**.

**FIGURE 4:**
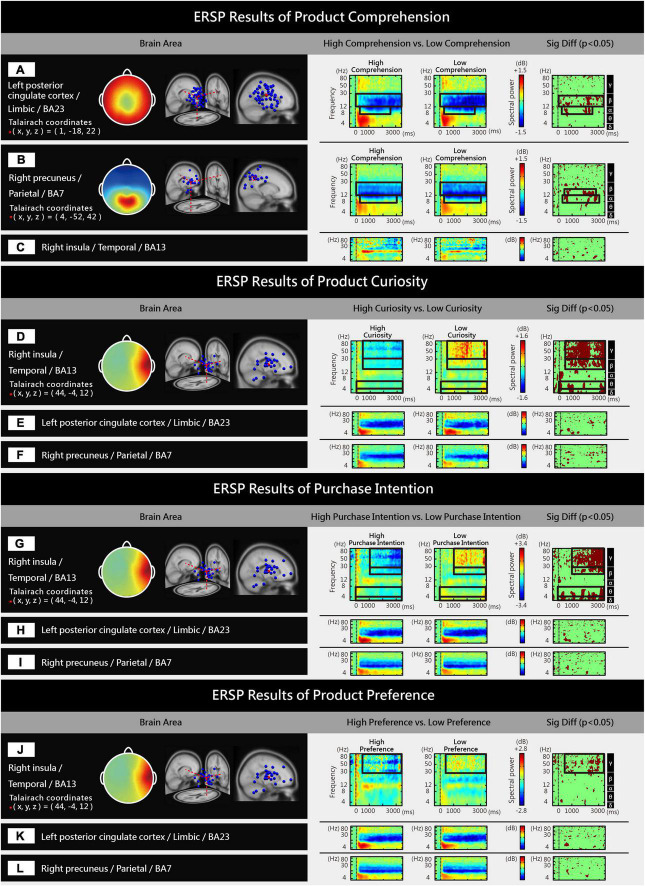
Significant differences were observed in the ERSPs of different brain areas between high/low levels of internal consumer responses: **(A,B)** High product comprehension led to higher spectral power in the left PCC and right precuneus than low product comprehension (*p* < 0.05). **(C)** No significant difference was found between high and low product comprehension in the spectral power of the right insula (*p* > 0.05). **(D)** High product curiosity led to lower spectral power in the right insula than low product curiosity (*p* < 0.05). **(E,F)** No significant difference was found between high and low product curiosity in the spectral power of the left PCC and right precuneus (*p* > 0.05). **(G)** High purchase intention led to lower spectral power in the right insula than low purchase intention (*p* < 0.05). **(H,I)** No significant difference was found between high and low purchase intention in the spectral power of the left PCC and right precuneus (*p* > 0.05). **(J)** High product preference led to lower spectral power in the right insula than low product preference (*p* < 0.05). **(K,L)** No significant difference was found between high and low product preference in the spectral power of the left PCC and right precuneus (*p* > 0.05).

**FIGURE 5:**
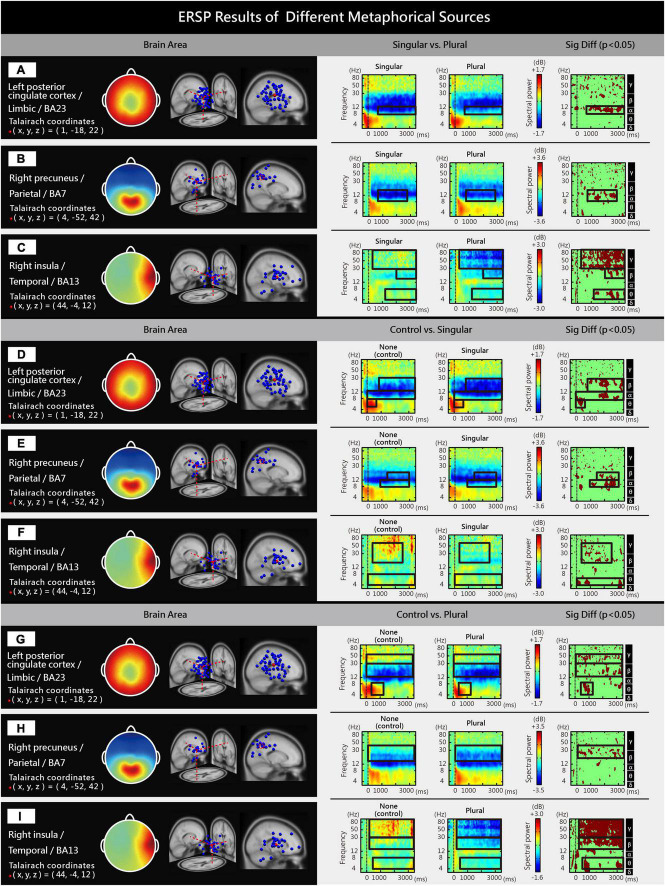
Significant differences were observed in the ERSPs of different brain areas among marketing images with different numbers of metaphorical sources: **(A,D,G)** In the left PCC, the spectral power of plural metaphorical sources was higher than that of singular metaphorical sources, while the spectral power of no metaphorical source was higher than that of both singular and plural metaphorical sources. **(B,E,H)** In the right precuneus, the spectral power of plural metaphorical sources was higher than that of singular metaphorical sources, while the spectral power of no metaphorical sources was higher than that of both singular and plural metaphorical sources. **(C,F,I)** In the right insula, the spectral power of singular metaphorical sources was higher than that of plural metaphorical sources, while the spectral power of no metaphorical sources was higher than that of both singular and plural metaphorical sources.

An independent components analysis (ICA) was performed on the 64-channel EEG signals of the 30 participants to obtain 1920 independent components (ICs). Based on the equivalent dipole location and scalp map features, K-means clustering was conducted to cluster the 1920 ICs into 13 brain regions (Figure 3). ESRP was employed to analyze high/low levels of internal consumer responses (Figure 4) and surreal marketing images with different numbers of metaphorical sources (Figure 5). Please refer to the research methods for a detailed description of ICA and ERSP analysis. Significant differences in spectral power were identified in three brain areas (*p* < 0.05), the left PCC [limbic region, Brodmann Area (BA) 23, Figure 3A], right precuneus (parietal region, BA7, Figure 3B), and right insula (temporal region, BA13, Figure 3C).

#### High product comprehension led to increased beta and alpha spectral power in the left PCC and right precuneus

Significant differences (*p* < 0.05) were found between high/low product comprehension in the beta (β, 14–30 Hz) and alpha (α, 8–13 Hz) spectral power of the left PCC and right precuneus, with high product comprehension eliciting greater levels than low product comprehension (Figures 4A,B). In the ERSP map of high product comprehension, yellow and red represent increased EEG spectral power. In the ERSP map of low product comprehension, blue represents decreased EEG spectral power. In the significant difference map, coffee color represents significant differences in spectral power between high/low product comprehension (*p* < 0.05), and a more clustered, more continuous, and larger coffee color area within a given region indicates a more significant difference in spectral power. There was no significant difference (*p* > 0.05) in spectral power between high/low product comprehension in the right insula (Figure 4C). The coffee-colored areas in the significant difference map were not clustered or continuous, implying that the difference in spectral power was not significant.

#### High product curiosity led to decreased gamma, beta, theta, and delta spectral power in the right insula

Significant differences (*p* < 0.05) were found between high/low product curiosity in the gamma (γ, 31–100 Hz), beta (β, 14–30 Hz), theta (θ, 4–7 Hz) and delta (δ, 1–3 Hz) spectral power of the right insula, with low product curiosity exhibiting greater levels than high product curiosity (Figure 4D). There was no significant difference (*p* > 0.05) in spectral power between high/low product curiosity in the left PCC and right precuneus (Figures 4E,F).

#### High purchase intention led to decreased gamma, beta, theta, and delta spectral power in the right insula

Significant differences (*p* < 0.05) were found between high/low purchase intention in the gamma (γ, 31–100 Hz), beta (β, 14–30 Hz), theta (θ, 4–7 Hz) and delta (δ, 1–3 Hz) spectral power of the right insula, with low purchase intention exhibiting greater levels than high purchase intention (Figure 4G). There was no significant difference (*p* > 0.05) in spectral power between high/low purchase intention in the left PCC and right precuneus (Figures 4H,I).

#### High product preference led to decreased gamma spectral power in the right insula

A significant difference (*p* < 0.05) was found between high/low purchase preference in the gamma (γ, 31–100 Hz) spectral power of the right insula, with low product preference eliciting a greater level than high product preference (Figure 4J). There was no significant difference (*p* > 0.05) in spectral power between high/low product preference in the left PCC and right precuneus (Figures 4K,L).

To summarize the ERSP results of internal consumer responses and high-level product comprehension led to consistent increases in the spectral power of the left PCC and right precuneus, whereas high-level product curiosity, purchase intention, and product preference led to consistent increases in the spectral power of the right insula. See Table 1 below.

#### Surreal marketing images with more metaphorical sources led to higher alpha spectral power in the left PCC

Significant differences (*p* < 0.05) were found among surreal marketing images with different numbers of metaphorical sources in the gamma (γ, 31–100 Hz), beta (β, 14–30 Hz), alpha (α, 8–13 Hz), and theta (θ, 4–7 Hz) spectral power of the left PCC. The plural metaphorical sources group showed a higher alpha spectral power than the singular metaphorical source group (Figure 5A). The control group showed higher beta, alpha, and theta spectral power than the singular metaphorical source group (Figure 5D). The control group showed higher gamma, beta, and theta spectral power than the plural metaphorical sources group (Figure 5G).

#### Surreal marketing images with more metaphorical sources led to higher beta and alpha spectral power in the right precuneus

Significant differences (*p* < 0.05) were found among surreal marketing images with different numbers of metaphorical sources in the beta (β, 14–30 Hz) and alpha (α, 8–13 Hz) spectral power of the right precuneus. Images with plural metaphorical sources evoked higher beta and alpha spectral power than those with a singular metaphorical source (Figure 5B). The control group showed higher beta and alpha spectral power than the singular metaphorical source group (Figure 5E). It also elicited a higher beta spectral power than the plural metaphorical sources group (Figure 5H).

#### Surreal marketing images with more metaphorical sources led to lower gamma, beta, and theta spectral power in the right insula

Significant differences (*p* < 0.05) were found among surreal marketing images with different numbers of metaphorical sources in the gamma (γ, 31–100 Hz), beta (β, 14–30 Hz) and theta (θ, 4–7 Hz) spectral power of the right insula. The singular metaphorical source images were associated with higher gamma, beta, and theta spectral power than the plural metaphorical source images (Figure 5C). The control group elicited higher gamma, beta, and theta spectral power than the singular metaphorical source group (Figure 5F). It also evoked higher gamma, beta, and theta spectral power than the plural metaphorical sources group (Figure 5I).

To summarize, surreal marketing images with a higher number of metaphorical sources (plural metaphorical sources) showed higher spectral power in the left PCC and right precuneus but lower spectral power in the right insula than surreal marketing images with singular metaphorical sources. Non-surreal marketing images with no metaphorical sources consistently showed higher spectral power in the left PCC, right precuneus, and right insula than surreal marketing images with singular or plural metaphorical sources.

## Discussion

**FIGURE 6:**
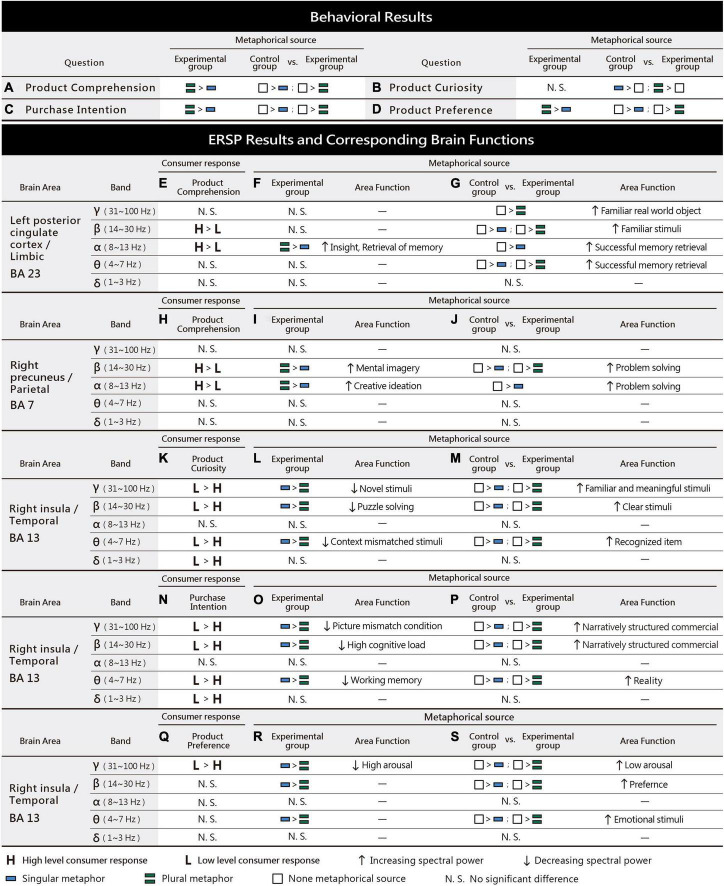
**(A–D)** Behavioral results showing the effects of different numbers of metaphorical sources on product comprehension, curiosity, intention, and preference. **(E–S)** ERSP results of high/low-level consumer responses, different metaphorical sources, and corresponding brain functions.

Figure 6 summarizes the behavioral (Figure 2) and ERSP results (Figures 4, 5). In this section, we will verify our research hypothesis that surreal marketing images with more metaphorical sources are likely to evoke greater EEG spectral perturbations of internal consumer responses (product curiosity, comprehension, preference, and purchase intention). The following is a discussion of the brain areas and frequency bands related to different internal consumer responses and different numbers of metaphorical sources.

### High product comprehension led to increased spectral power in the left PCC: Association with insight and episodic memory retrieval

Surreal marketing images with high product comprehension and more metaphorical sources evoked greater alpha spectral power in the left PCC (Figures 6E,F). Behavioral results revealed that the product comprehension of the plural metaphorical sources group was higher than that of the singular metaphorical source group (Figure 6A). The PCC is part of the limbic system (Leech and Sharp, 2014), and the functions of the left PCC are associated with insight (Jung-Beeman et al., 2004). Insight refers to a type of sudden understanding, which encompasses the swift comprehension of a metaphor or the sudden recognition of objects in ambiguous images (Bowden and Jung-Beeman, 2007; Kounios and Beeman, 2009; Sprugnoli et al., 2017). When performing insight-related tasks that require associative thinking, participants must consider the widest possible range of different reasons to explain an unusual situation, which will lead to an alpha-band increase in the posterior part of the brain (Fink et al., 2007). In this study, surreal marketing images with more metaphorical sources, such as an image of a mouthwash product with plural metaphorical sources, meant that participants had to consider different possible reasons which might explain the unusual context that combined onions, mouths, and the mouthwash product. The non-surreal marketing images with no metaphorical source in the control group evoked higher gamma, beta, alpha, and theta spectral power in the left PCC (Figure 6G). Behavioral results showed that the production comprehension of no metaphorical source images was higher than that of both the singular and plural metaphorical source images (Figure 6A). PCC function is also related to episodic memory retrieval (Sestieri et al., 2011; Vanneste et al., 2021), and this area can be activated by familiar objects and scenes (Sugiura et al., 2005). Episodic memory serves to recall and store previous experiences or episodes, and to link current knowledge with past experiences (Lundstrom et al., 2003). Participants exhibit an increase in the gamma band power of the PCC during episodic memory retrieval (Lega et al., 2017), and familiar real-world objects may induce an early increase in the gamma power of posterior electrodes (Busch et al., 2006). Familiar stimuli evoked higher beta-band activity in the central region compared to unfamiliar stimuli (Tanaka and Kudo, 2012). Furthermore, successfully retrieved images elicited PCC activation and increases in alpha and theta power (Herweg et al., 2016). In this study, actual product images without metaphorical sources tended to depict the original appearance and features of familiar and concrete products, such as watches, cameras and cars, which enabled the participants to successfully link current products with past experiences.

### High product comprehension led to increased spectral power in the right precuneus: Association with imagination and creative thinking

Surreal marketing images with high product comprehension and more metaphorical sources evoked greater beta and alpha spectral power in the right precuneus (Figures 6H,I). Behavioral results indicated that the product comprehension of the images with plural metaphorical sources was higher than that of images with a singular metaphorical source (Figure 6A). The precuneus is in the parietal region of the brain, and its functions are related to mental imagery processing (Cavanna and Trimble, 2006; Mashal et al., 2014). Mental imagery refers to images conceived only in the mind (Jia et al., 2021). Participants exhibited an increase in the beta-band activity of the parietal region when thinking of various images (Villena-González et al., 2018). During creative ideation, an increase in alpha power was observed in the right parietal region. Creative ideation refers to the process of producing several different original ideas to solve an open problem (Fink and Benedek, 2014). In this study, surreal marketing images with several metaphorical sources (plural metaphorical sources) required creative ideation to connect these metaphors with the product design and function. The non-surreal marketing images with no metaphorical sources in the control group evoked higher beta and alpha spectral power in the right precuneus (Figure 6J). Behavioral results indicated that the product comprehension of images with no metaphorical sources was higher than that of images with singular and plural metaphorical sources (Figure 6A). Related studies on puzzle-solving have found that participants who are able to solve more puzzles exhibit increased beta power in the parietal region (Kounios et al., 2008), although increased alpha power is also observed in this region during successful problem solving (Cao et al., 2015). In this study, actual product images without metaphorical sources directly depicted the product’s true features and functions, which led easily to successful comprehension of the marketing images.

### High product curiosity led to decreased spectral power in the right insula: Association with puzzle-solving and novelty

Surreal marketing images with high product curiosity and more metaphorical sources evoked lower gamma, beta, and theta spectral power in the right insula (Figures 6K,L). Behavioral results suggest that the product curiosity inspired by images with singular metaphorical sources and those with plural metaphorical sources was higher than those of no metaphorical sources (Figure 6B). The insula, a small cortical region buried under the temporal region (Zurawicki, 2010; Ward, 2016), is involved in the processing of curiosity (Jepma et al., 2012; Van Lieshout et al., 2018). Participants in the high-curiosity condition exhibited greater insula activation than those in the low-curiosity condition (Wiggin et al., 2018). The insula is also activated during the puzzle-solving process (Aziz-Zadeh et al., 2009; Sprugnoli et al., 2017). Novel stimuli evokes lower gamma activity in the insula than familiar stimuli (Citherlet et al., 2019), and the beta power of the temporal region is reduced during puzzle-solving processes (Sheth et al., 2009). Hence, participants produced a smaller high theta power in the context-mismatch condition between word-movie pairs compared to the context-match condition (Staudigl and Hanslmayr, 2013). This is similar to samples with plural metaphorical sources in our study, in which unusual, mismatched images of a human hand and a wrench were merged to make a connection with hand-made cars. Non-surreal marketing images with no metaphorical source evoked higher gamma, beta, and theta spectral power in the right insula (Figure 6M). Behavioral results indicated that product curiosity associated with images with no metaphorical sources was lower than that linked to images with singular and plural metaphorical sources (Figure 6B). Participants exhibited higher gamma band activity in the temporal region when viewing meaningful and familiar objects than meaningless and unfamiliar objects (Gruber et al., 2008). Clear images elicited greater beta power in the anterior insula than noisy images (Chand and Dhamala, 2016), while old pictures recognized by participants evoked greater theta activity in the right parietotemporal areas than new pictures (Osipova et al., 2006). In this study, real marketing images without metaphorical sources depicted the true appearances and features of familiar products, and, therefore, were clear and recognizable.

### High purchase intention led to decreased spectral power in the right insula: Association with cognitive load and craving

Surreal marketing images with high purchase intention and more metaphorical sources evoked lower gamma, beta, and theta spectral power in the right insula (Figures 6N,O). Behavioral results revealed that the purchase intention evoked by images with plural metaphorical sources was higher than those of images with a singular metaphorical source (Figure 6C). The insula is involved in the reward system (Bonson et al., 2002; Villafuerte et al., 2012), and changes in its activation can be used to predict subsequent product purchases (Knutson et al., 2007; Tusche et al., 2010). Some have observed that difficult decision-making conditions, in which categories of objects are difficult to detect, lead to higher insula modulation (Castelhano et al., 2014). Incongruous images elicit an early decrease in gamma power (Willems et al., 2008). Tasks with high cognitive load cause decreases in beta power across extensive brain areas. When performing a multi-object tracking task, participants showed a higher cognitive load when asked to track more targets (Boring et al., 2020). Theta activity is also decreased under a stressful working memory load (Gärtner et al., 2014). Studies have shown that self-regulatory depletion can induce cravings for reward (Wagner et al., 2013), whereas cognitive depletion can decrease the resistance to temptation (Fedorikhin and Patrick, 2010), for example, by leading to impulse buying (Vohs and Faber, 2007). The right insula plays a role in the impulse to take risks (Xue et al., 2010), and drug addicts exhibit insula activation during their impulse to take drugs (Naqvi and Bechara, 2009). In this study, surreal marketing images with plural metaphorical sources contained more targets that were more difficult to detect. Non-surreal images with no metaphorical source evoked higher gamma, beta, and theta spectral power in the right insula (Figure 6P). Behavioral results showed that the purchase intention associated with images with no metaphorical source was higher than that of images with singular and plural metaphorical sources (Figure 6C). Narratively structured video commercials, with a full storyline, clear communication, and greater meaningfulness, produce higher gamma and beta power in the right temporal region (Wang et al., 2016). In a virtual environment, higher theta activation in the insula is associated with a greater sense of presence (Clemente et al., 2014). In this study, the actual product images with no metaphorical source depicted the true appearances and features of the product. These were associated with a low cognitive load, and presented a full narrative structure regarding the product design and function.

### High product preference led to decreased spectral power in the right insula: Association with emotional choices

Surreal marketing images with high product preference and more metaphorical sources evoked lower gamma spectral power in the right insula (Figures 6Q,R). Behavioral results indicated that the product preferences evoked by plural metaphorical sources was higher than that evoked by singular metaphorical sources (Figure 6D). The insula is involved in preference judgments (Chaudhry et al., 2009) and emotional processing (Gogolla, 2017). High arousal stimuli elicit lower gamma activity in the right brain, and gamma activity is believed to reflect the enhancement of subjective motivation when describing stimuli (Balconi and Pozzoli, 2009). The high visual complexity of surreal images provide high arousal stimuli (Briggs and Martin, 2009), and the surreal marketing images with plural metaphorical sources used in this study were stimuli with relatively high visual complexity. We found that product images with no metaphorical sources in the control group led to higher gamma, beta, and theta spectral power in the right insula (Figure 6S). Behavioral results showed that the product preferences of the control group were higher than those of the singular and plural metaphorical sources (Figure 6D). Compared to neutral brands that participants neither liked nor disliked, enhanced activation of the insula was observed for brands with which participants had established close relationships (e.g., Starbucks or Disney), and which involved more subjective and emotional choice evaluations (Reimann et al., 2012). Participants who viewed images of their preferred crackers exhibited higher beta power in the temporal region superior to the insula (Khushaba et al., 2013). Emotional stimuli (e.g., happy or angry faces) evoked higher theta power in the insula than neutral stimuli (Knyazev et al., 2009). In this study, the actual product images without metaphorical sources that were presented were usually to be found in real life and had a closer relationship with participants, compared to the surreal images with plural metaphorical sources.

## Conclusion

Our study findings have verified that surreal marketing images eliciting different internal consumer responses led to significant differences in the gamma, beta, alpha, and theta spectral power of the left PCC, right precuneus, and right insula. Surreal marketing images with high product comprehension evoked higher beta and alpha spectral power in the left PCC and right precuneus, which are involved in insight, memory retrieval, mental imagery processing, and creative ideation. Surreal marketing images with high product curiosity and high purchase intention exhibited consistent trends in spectral power, evoking lower gamma, beta, and theta spectral power in the right insula, which are involved in novelty, puzzle-solving, and cravings for reward caused by cognitive overload. Surreal marketing images with high product preference evoked lower gamma spectral power in the right insula, which is involved in emotional choice. It is suggested that further studies conduct comparative explorations of consumers’ curiosity affecting their willingness to buy, using higher-level suitable neuroscience tools such as fMRI.

## Data availability statement

The raw data supporting the conclusions of this article will be made available by the authors, without undue reservation.

## Ethics statement

The studies involving human participants were reviewed and approved by the Institutional Review Board of Cathay General Hospital (IRB number: CGH-TECH106001). The patients/participants provided their written informed consent to participate in this study.

## Author contributions

RW designed the experiment and interpreted the results, in cooperation with I-NL. I-NL performed the data analysis. Both authors wrote the manuscript accordingly.
